# Do we really need to panic in all anisocoria cases in critical care?

**DOI:** 10.4103/0019-5049.68398

**Published:** 2010

**Authors:** Saban Yalcin, Kutluk Pampal, Aydin Erden, Sirali Oba, Selçuk Bilgin

**Affiliations:** Department of Anaesthesiology and Reanimation, Harran University Medical Faculty, Turkey

Sir,

Anisocoria (a unilateral dilated pupil) in critical care patients is a point of concern, which warrants a thorough examination and often, also, costly investigations to rule out a serious cause.[1]

**Figure 1:**
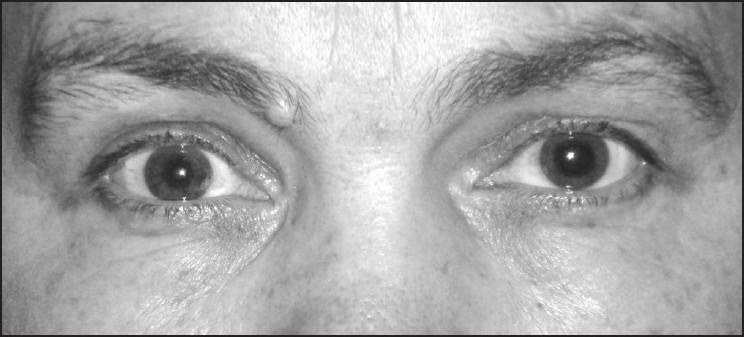
Right pupil was both normal in size and in responsiveness to light, and her left pupil was fully dilated after nebulised albuterol (salbutamol) and ipratropium bromide through a face mask

A 48-year-old female patient transferred to our intensive care for further management of respiratory failure due to fungal pneumonia. After 12 hours from admission, her nurse noticed that, her right pupil was both normal in size and in responsiveness to light, and her left pupil was fully dilated [Figure 1]. Examination of the cranial nerves showed no other abnormalities. A computed tomographic scan of her head, performed to search for an intracranial cause of partial palsy of the third cranial nerve, was normal. Further, review disclosed that she received oxygen and nebulised albuterol (salbutamol) and ipratropium bromide through a face mask. The face mask was found to fit imperfectly and leak slightly to the left. The anisocoria resolved within 24 hours after the patient stopped receiving ipratropium.

Anisocoria, or unequal pupil size, may be an early sign of an impending neurologic emergency in any patient and often suggestive of a life threatening condition affecting cranial nerve function, such as tumour compression, intracranial hypertension with impending uncal herniation, expanding intracranial aneurysm, or haemorrhage. Benign mydriasis can be due to prior trauma, medication effects, and congenital abnormalities. Determining the cause of anisocoria can be challenging in critical care settings because patients often are sedated, paralysed, intubated, or have a baseline altered mental status that makes full neurologic examination difficult. The workup of acute anisocoria frequently involves costly and/or invasive procedures, including computed tomography (CT), magnetic resonance imaging, electroencephalography, lumbar puncture, and neurologic consultations before ruling out the most serious causes.[2]

Ipratropium bromide is a quaternary amine derivative of atropine and a direct antagonist at muscarinic cholinergic receptors and it was not considered a cause of anisocoria until 1986 when Samaniego and Newman described the first case.[3] Contamination of the eye from nebulized ipratropium bromide leads to asymmetric pupillary dilation by paralyzing the parasympathetic nerve endings. The anisocoria, which is usually resolves within 48 hours of removal of the agent, sometimes may last up to three weeks after the aerosolised bronchodilator is stopped. Other manifestations of ipratropium exposure include bilateral mydriasis, cycloplegia, blurred vision, dry eyes, and in rare cases, acute glaucoma. Failure of the dilated pupil to constrict after instillation of 1% of pilocarpine hydrochloride confirms the diagnosis. Ipratropium bromide should be considered in the differential diagnosis of patients with anisocoria when no structural explanation can be found with a brain CT.[4,5]
